# Effect of Drying Methods on Bioactivity of *Pyrostegia venusta* Extracts: Antioxidant Assays, Cytotoxicity, and Computational Approaches

**DOI:** 10.3390/ph18091315

**Published:** 2025-09-02

**Authors:** Milena Cremer de Souza, Letícia Bertini, Julia Estrella Szmaruk, Matheus Ribas de Almeida, Maria Luisa G. Agneis, Roberta Carvalho Cesário, Wesley Ladeira Caputo, Christiane Luciana da Costa, Vitor Augusto dos Santos Garcia, Fábio R. F. Seiva

**Affiliations:** 1 Department of Chemistry and Biochemistry, Institute of Bioscience, São Paulo State University (UNESP), Botucatu 18618-686, SP, Brazil; milena.cremer@unesp.br (M.C.d.S.); julia.szmaruk@unesp.br (J.E.S.); matheus.ribas-almeida@unesp.br (M.R.d.A.); maria.agneis@unesp.br (M.L.G.A.); roberta.cesario@unesp.br (R.C.C.); wesley.l.caputo@unesp.br (W.L.C.); 2 Biological Science Center, North of Paraná State University (UENP), Bandeirantes 86360-000, PR, Brazil; lehber1998@gmail.com (L.B.); christiane@uenp.edu.br (C.L.d.C.); 3 Department of Vegetable Production, Faculty of Agricultural Sciences, São Paulo State University (UNESP), Botucatu 18610-034, SP, Brazil; vitor.as.garcia@unesp.br

**Keywords:** phenolic compounds, plant extracts, cell proliferation, molecular docking

## Abstract

**Background/Objectives: ***Pyrostegia venusta* (Cipó-de-São-João), a native Brazilian Cerrado plant, is rich antioxidant phytochemicals. The efficacy of herbal extracts, particularly their phenolic content and antioxidant potential, is influenced by the extraction method used. This study investigated the effects of two drying methods, hot-air oven drying and freeze-drying, on the antioxidant activity, cytotoxicity, and molecular interactions of aqueous extracts from the flowers and leaves of *P. venusta*. **Methods:** antioxidant capacity was assessed using DPPH, FRAP, and Folin–Ciocalteu assays; phenolic profiles were characterized by UHPLC; and cytotoxicity was evaluated via the MTT assay in HaCaT human keratinocyte cells. Additionally, in silico ADMET predictions were conducted to assess pharmacokinetics and potential toxicity, followed by molecular docking to evaluate interactions with the proliferation markers Ki-67 and PCNA. **Results:** freeze-dried extracts, particularly from the flowers, contained higher concentrations of phenolic compounds and exhibited superior antioxidant activity compared to hot-air oven-dried extracts. UHPLC analysis identified a range of bioactive phenolics including caffeic, chlorogenic, gallic, ferulic, and p-coumaric acids, quercetin, and anthocyanidins such as pelargonidin-3-O-glucoside and peonidin-3-O-glucoside, with distinct compositional differences between leaves and flowers. ADMET analysis revealed generally favorable pharmacokinetic properties for most compounds. Docking simulations indicated that multiple phenolics showed synergistic interactions with Ki-67 and PCNA. **Conclusions:** our findings highlight freeze-drying as the optimal method for preserving bioactive compounds in *P. venusta* and support the therapeutic potential of its flower extracts. The evidence supports the notion that the biological effects of *P. venusta* are driven by synergism among multiple constituents rather than isolated compounds.

## 1. Introduction

Medicinal plants have been extensively utilized throughout human history as primary therapeutic agents due to their accessibility, affordability, and broad pharmacological potential, which, in some cases, surpass those of conventional drugs [1]. Phytotherapeutics, bioactive compounds derived from medicinal plants, have demonstrated significant therapeutic properties that have been explored in multiple medical contexts, including fungal and bacterial infections, cardiovascular diseases, cancer, and diabetes. Among these, polyphenols stand out due to their antioxidant capacity and minimal adverse effects, being available in diverse forms such as extracts, essential oils, and whole or fragmented plant materials [2,3,4,5,6].

*Pyrostegia venusta*, commonly known as Cipó de São João, is a climbing plant native to the Brazilian Cerrado and belongs to the Bignoniaceae family. Traditionally, it has been employed in the treatment of colds, flu, diarrhea, and vitiligo [7]. The plant is particularly rich in phenolic compounds, recognized for their capacity to neutralize free radicals and protect cellular structures, such as proteins, membranes, and DNA, from oxidative damage [8,9]. The antioxidant activity of plant extracts is strongly influenced by their phytochemical composition [10], which can vary based on the plant organ used (e.g., leaf, flower, or root), the type of solvent employed, and the drying and extraction methods adopted. Solvent selection is particularly critical, as solvents with different polarities can affect the composition and yield of the extracted bioactive compounds. Similarly, drying techniques, including freeze-drying and forced-air oven drying, can alter the final composition of phenolic constituents [11,12].

Previous studies have identified a variety of bioactive constituents in *P. venusta*. Flowers are known to contain carotenoids, xanthophylls, oleanolic acid, β-sitosterol, β-amyrin, stigmasterol, and n-hentriacontane [13,14], whereas leaves contain alkaloids, flavonoids, diterpenes, and saponins [15]. Ethanolic and methanolic extracts of *P. venusta* have demonstrated anti-inflammatory and antioxidant activities, with no reported toxicity in animal models [16,17,18]. However, these previous investigations relied on organic solvents, and little is known about the composition and biological effects of aqueous extracts, particularly from leaves and fruits.

The extraction of polyphenols using water as a solvent has gained prominence as a sustainable, effective, and eco-friendly alternative to conventional organic solvents such as ethanol and methanol. From a green chemistry perspective, water is a desirable solvent due to its non-toxic, renewable, and biocompatible nature, especially for applications in the pharmaceutical and nutraceutical industries [19]. Aqueous extraction not only avoids toxic residues, but also enhances the recovery and stability of water-soluble polyphenols, potentially improving their bioavailability and efficacy. Recent advancements in green extraction techniques have further supported this approach by minimizing solvent use and energy consumption while preserving thermolabile bioactive compounds [19,20,21].

Natural plant extracts have also shown the ability to modulate cellular proliferation, promoting or inhibiting cell growth depending on the biological context [22,23]. Among the key markers of cell proliferation are Ki67 and PCNA (Proliferating Cell Nuclear Antigen). PCNA functions as a sliding clamp and processivity factor in DNA replication, interacting with DNA polymerase δ, and plays a critical role in DNA metabolism [24], while Ki67, encoded by the MKI67 gene (Marker of Proliferation Ki-67), is expressed during all active phases of the cell cycle (G1, S, G2, and M) but is absent in quiescent cells (G0), making it a widely accepted proliferation marker [25]. Physiologically, both proteins are indispensable for tissue growth, regeneration, and homeostasis. Their expression is upregulated in healthy proliferating cells, while aberrant overexpression of Ki67 and PCNA is often associated with cancer [26,27]. Thus, controlled activation of these proteins could be advantageous in therapeutic contexts where enhanced cell proliferation is desired, such as in wound healing, skin repair, neurodegeneration, and degenerative diseases.

In this context, molecular docking has emerged as a valuable computational strategy to investigate the interaction between phytochemicals and biological targets. This in silico approach enables rapid, cost-effective screening of complex plant extracts by predicting binding affinities and interaction modes with proteins of interest [28,29]. Such tools are particularly advantageous for identifying novel bioactive candidates from plant sources, supporting the growing interest of the pharmaceutical industry in natural-product-based therapeutics.

Considering the influence of extraction strategy on the phytochemical profile, the lack of data regarding aqueous extracts of different *P. venusta* organs, and the relevance of evaluating their biological effects, this study aimed to characterize extracts obtained via distinct methods. Specifically, we identified phenolic constituents, assessed antioxidant activity in vitro, and evaluated cytotoxicity in HaCaT human keratinocytes. Additionally, molecular docking was performed to explore potential interactions between selected phytochemicals and the proliferation-associated proteins Ki67 and PCNA.

## 2. Results

### 2.1. Freeze-Dried Flower Extract Exhibited Higher Concentration of Phenolic Compounds, Antioxidant Capacity, and Enhanced HaCaT Cell Viability

**Figure 1:**
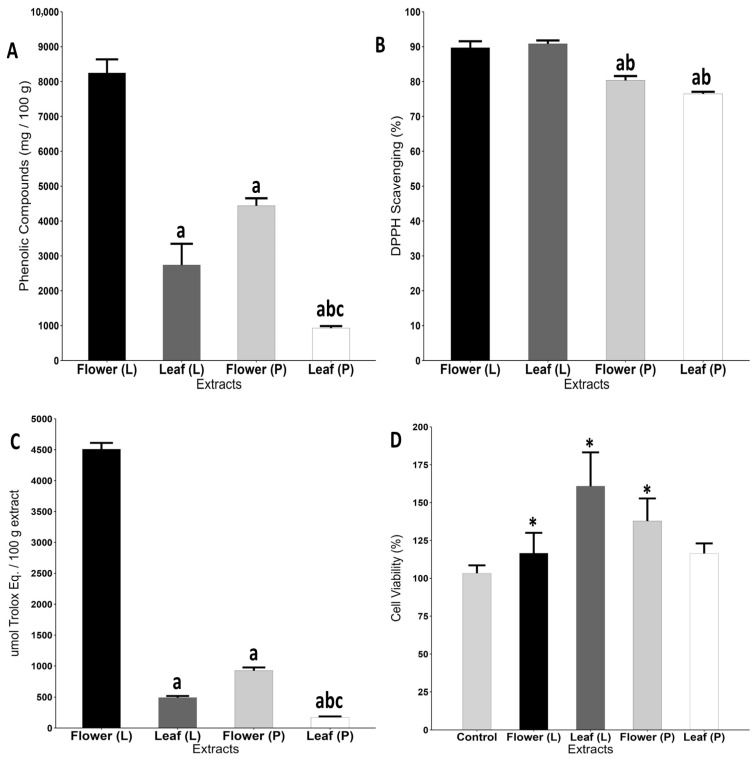
Antioxidant and cytotoxicity profile of hot-air oven-dried (P) and lyophilized (L) extracts of *Pyrostegia venusta*. (**A**): Concentration of total phenolic compounds; (**B**): antioxidant capacity (DPPH reduction); (**C**): antioxidant activity (FRAP assay); and (**D**): cell viability of Hacat cells accessed by the MTT assay. Data were analyzed by One-Way ANOVA complemented with Tukey’s test for multiple comparisons and expressed as the mean ± SEM. Letters: a: differ statistically from flower (L) group; b: differ statistically from leaf (L) group; c: differ statistically from flower (P) group; *: differ statistically from control group.

The total phenolic content of the extracts was quantified using the Folin–Ciocalteu method. Among the tested samples, the freeze-dried flower extract showed the highest concentration of phenolic compounds. In contrast, the hot-air oven-dried leaf extract presented the lowest concentration, being 93% lower than the freeze-dried flower (*p* < 0.0001) and 88% lower than the freeze-dried leaf (*p* = 0.0478). The hot-air oven-dried flower extract also showed a significant reduction, with a 55% lower phenolic content compared to the freeze-dried flower (*p* = 0.0478) (Figure 1A).

Antioxidant capacity and activity were assessed using the DPPH and FRAP assays, respectively. The freeze-dried flower and freeze-dried leaf extracts displayed a similarly high antioxidant capacity. In comparison, the hot-air oven-dried flower extract exhibited a significantly lower antioxidant capacity than the freeze-dried leaf (*p* = 0.0364) and flower (*p* = 0.0361) extracts. A similar trend was observed for the hot-air oven-dried leaf extract, which also showed a reduced antioxidant capacity compared to the freeze-dried leaf (*p* = 0.0002) and flower (*p* = 0.0002) extracts (Figure 1B).

In terms of antioxidant activity, the freeze-dried flower extract demonstrated the highest values. Both the hot-air oven-dried flower and freeze-dried leaf extracts showed significantly lower activity (*p* < 0.0001 for both). The hot-air oven-dried leaf extract showed the lowest antioxidant activity overall, with statistically significant differences when compared to the freeze-dried flower (*p* < 0.0001), freeze-dried leaf (*p* = 0.0095), and hot-air oven-dried flower (*p* = 0.0003) extracts (Figure 1C).

The MTT cytotoxicity assay was performed to assess the impact of the extracts on HaCaT cell viability. Interestingly, rather than reducing viability, all extracts promoted a significant increase compared to the control group. The hot-air oven-dried flower extract led to a 57% increase in cell viability (*p* < 0.0001), followed by the freeze-dried flower extract with a 36% increase (*p* = 0.0226) and the hot-air oven-dried leaf extract with a 32% increase (*p* = 0.0281) (Figure 1D).

### 2.2. UHPLC Analysis of Phytochemical Compound Profiles

Given that the lyophilized extracts exhibited the highest concentrations of phenolic compounds, they were selected for compound identification and quantification via UHPLC (Table 1). The following phenolic compounds were identified in the freeze-dried flowers and leaves of *P. venusta*: *trans*cinnamic acid, gallic acid, *trans*ferulic acid, chlorogenic acid, caffeic acid, P-coumaric acid, 3-O-methyl-quercetin, luteolin, quercetin, pelargonidin-3-O-glucoside, malvidin-3-5-diglycoside, peonidin-3-O-glucoside, and mal-vidin-3-O-glucoside. Among these, chlorogenic and caffeic acids were detected in significantly higher concentrations in the leaves (*p* < 0.0001), whereas 3-O-methylquercetin, p-coumaric acid, malvidin-3-O-glucoside (*p* < 0.0001), and malvidin-3,5-diglucoside (*p* = 0.0172) were more abundant in the flowers. The remaining compounds were present at similar concentrations in both the freeze-dried flowers and leaves.

### 2.3. Structural and Biological Properties of Phenolic Compounds and Their Predicted Toxicity

Considering that bioactive compounds are primarily responsible for the pharmacological properties of herbal extracts, we analyzed the predicted biological and toxicological profiles of the identified phytochemicals. Initially, the polyphenols were evaluated for compliance with Lipinski’s Rule of Five, a key indicator of oral bioavailability. With the exception of Malvidin-3-O-glucoside, Malvidin-3,5-diglycoside, and Peonidin-3-O-glucoside, all other identified phytoconstituents exhibited no more than five hydrogen bond donors, ten hydrogen bond acceptors, a molecular weight below 500 Daltons, and a calculated octanol–water partition coefficient (LogP) not exceeding 5 (Table 2). These results indicate that most polyphenols in *P. venusta* extracts present favorable drug-likeness and pharmacokinetic properties.

**Figure 2:**
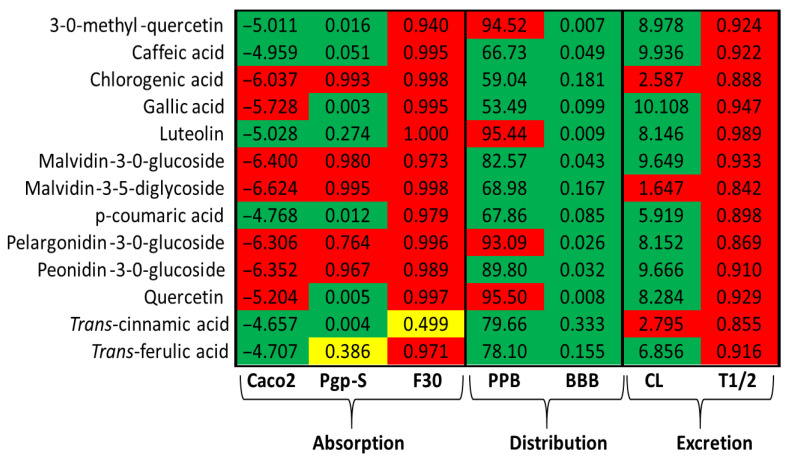
ADMET evaluation of phenolic compounds identified on leaves and flowers of *P. venusta*. Caco2: Probability of being absorbed by human colon adenocarcinoma cell; Pgp-S: probability of being a P-glycoprotein substrate; F30: probability of being bioavailable by oral route; PPB: probability of binding Plasma protein; BBB: probability of cross the blood–brain barrier; CL: probability of has high or low clearance rate; T1/2: probability of having a high or low half-life. All information was retrieved directly from the website (https://admetmesh.scbdd.com/explanation/index; accessed on 4 April 2025).

Further pharmacokinetic assessment was conducted using ADMET (Absorption, Distribution, Metabolism, Excretion, and Toxicity) predictions, summarized in Figure 2. Compounds such as 3-O-methylquercetin, luteolin, caffeic acid, p-coumaric acid, *trans*-cinnamic acid, and *trans*-ferulic acid demonstrated a good intestinal permeability and are predicted substrates for P-glycoprotein *trans*port. Among these, only *trans*-cinnamic acid exhibited a moderate oral bioavailability (~30%). These compounds also showed a high plasma protein binding affinity and are predicted to cross the blood–brain barrier (BBB). Furthermore, with the exception of *trans*-cinnamic acid, all compounds presented high predicted clearance rates and relatively short half-lives.

Given that hepatic metabolism is a critical pathway for orally administered herbal extracts, the potential for hepatotoxicity was also evaluated. Among the compounds tested, *trans*-cinnamic acid was the only one predicted to have a moderate hepatotoxic potential. Regarding other toxicological parameters, 3-O-methyl-quercetin, luteolin, and quercetin were predicted to be active for carcinogenicity, immunogenicity, mutagenicity, and/or nutritional toxicity. However, none of the analyzed polyphenols were predicted to exhibit toxicity across all evaluated parameters. Specifically, caffeic, chlorogenic, gallic, p-coumaric, *trans*-cinnamic, and *trans*-ferulic acids, along with pelargonid-in-3-O-glucoside, were identified as active for only one of the assessed toxicity parameters (Table 3).

### 2.4. Phenolic Compounds Interacted with Cell-Cycle Proteins

Molecular docking is an effective approach for identifying potential ligands that can bind to target proteins. In this study, we performed binding analyses of 12 polyphenols identified from the leaves and flowers of *P. venusta* with two well-known proteins involved in cell proliferation, Ki67 and PCNA. The results showed that all polyphenols exhibited a relatively high affinity for both proteins. Specifically, Malvidin-3-O-glucoside, Pelargonidin-3-O-glucoside, Peonidin-3-O-glucoside, Chlorogenic acid, Quercetin, and its analogue, 3-O-methyl-quercetin, demonstrated binding affinity energies lower than -10 kcal/mol to PCNA, with predicted inhibition constants ranging from 1.37 nmol/L to 83.04 µmol/L (Table 4). For Ki67, both Malvidin compounds, Peonidin-3-O-glucoside, 3-O-methyl-quercetin, and Pelargonidin-3-O-glucoside exhibited the lowest binding affinity energies, with calculated inhibition constants varying between 6.16 nmol/L and approximately 30 nmol/L.

**Figure 3:**
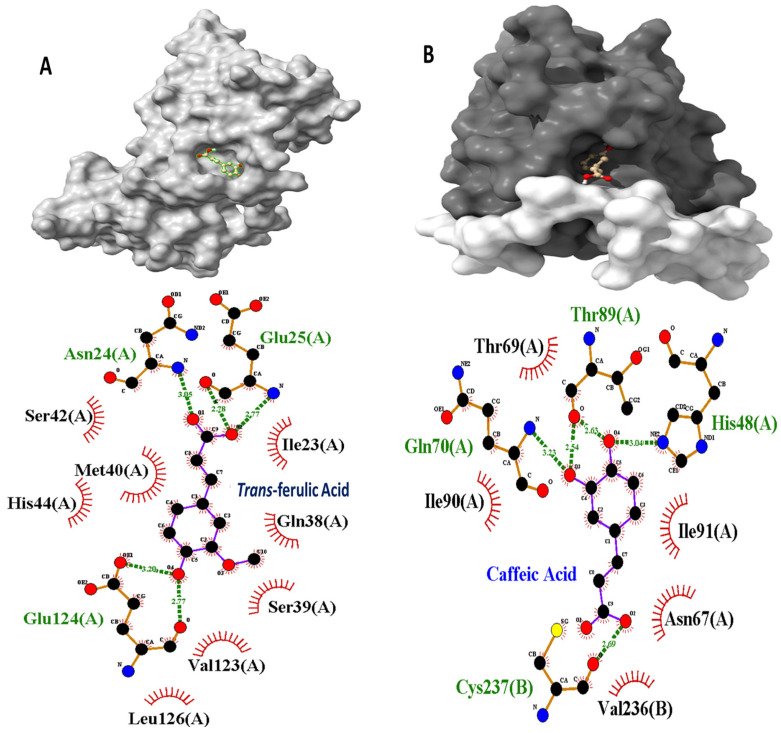
Docking poses and interactions of phenolic compounds identified in leaves and flowers of *P. venusta*. (**A**): main interactions between *trans*-ferulic acid and PCNA amino acid residues and (**B**): main interactions between caffeic acid and Ki-67 amino acid residues.

Given their favorable structural and pharmacokinetic properties, caffeic acid and *Trans*-ferulic acid were selected for a more detailed interaction analysis with PCNA and Ki67, respectively. *Trans*-ferulic acid was found to occupy a central pocket in PCNA, forming five hydrogen bonds with the residues Asn(24), Glu(25), and Glu(124). Additionally, the acid was stabilized through various hydrophobic interactions with residues such as Ile, Gln, Ser, Val, Leu, Met, and His (Figure 3A). Caffeic acid bound to Ki67, forming five hydrogen bonds with the amino acids His(48), Gln(70), Thr(89), and Cys(237) from the B subunit. These interactions were further supported by hydrophobic contacts with the residues Ile, Asn, Val, and Threonine (Figure 3B).

**Figure 4:**
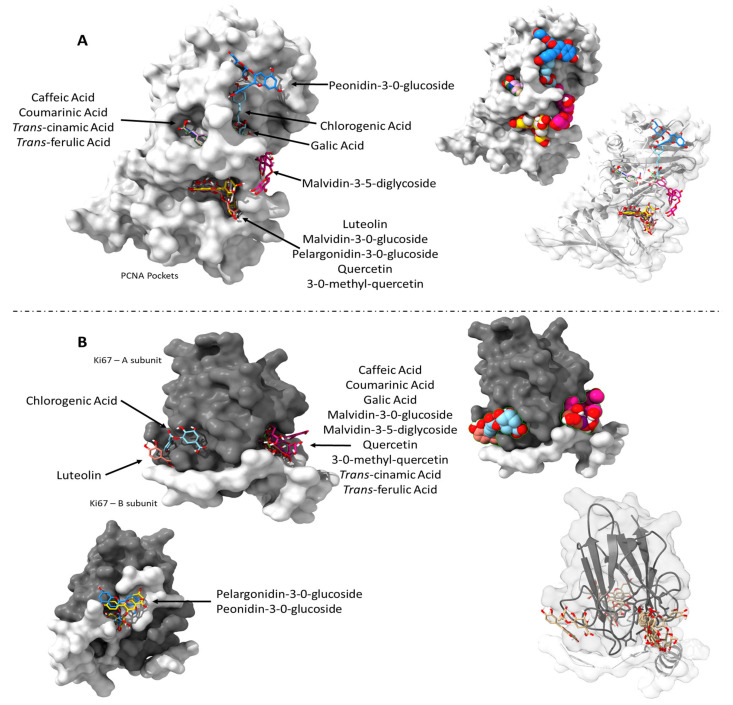
Docking poses and interactions of 13 phenolic compounds identified in leaves and flowers of *P. venusta*. (**A**): Binding sites of phenolic compounds within PCNA and (**B**): binding sites of phenolic compounds within Ki-67.

Lastly, we checked how all the phytochemicals identified in the extracts interacted with PCNA and Ki-67. When evaluating these interactions, it was observed that many of the compounds shared common binding sites (Figure 4). However, certain polyphenols, including Malvidin-3,5-diglycoside, Peonidin-3-O-glucoside, Chlorogenic acid, and Gallic acid, interacted with distinct pockets within the PCNA structure (Figure 4A). A similar pattern was observed for Ki67, where compounds such as Pelargonidin-3-O-glucoside and Peonidin-3-O-glucoside bound to a relatively distant site compared to other polyphenols (Figure 4B).

## 3. Discussion

The increasing use of natural products for the prevention and treatment of various human diseases has drawn significant attention to phytochemicals as safer and potentially more effective alternatives to synthetic drugs [30]. However, it is critical to recognize that the methods employed for the preparation of plant extracts can markedly influence their chemical composition and, consequently, their biological activity. Among the most common preparation techniques are shade-, oven- and freeze-drying methodologies. Oven drying involves the application of elevated temperatures, which can degrade or structurally alter thermolabile compounds, potentially diminishing the efficacy of the resulting extracts. In contrast, freeze-drying (lyophilization) removes approximately 98% of the water content under low-temperature and vacuum conditions, thereby better preserving the structural integrity and bioactivity of phytoconstituents. While oven drying typically removes only 70–80% of water and may leave residual moisture that promotes degradation, freeze-drying also reduces microbial proliferation and enzymatic activity, contributing to increased extract stability and shelf life [31,32]. These physicochemical advantages likely explain the significantly higher concentrations of phenolic compounds observed in the freeze-dried extracts compared to the oven-dried leaf and flower samples. This preservation is particularly relevant given the well-documented antioxidant, anti-inflammatory, and chemopreventive properties of phenolic compounds. The solvent used during extraction is another critical factor influencing the phytochemical profile of plant extracts. Methanolic extracts of *P. venusta* leaves have been reported to contain a higher phenolic content compared to those obtained with non-polar (hexane) or moderately polar (ethyl acetate) solvents. In the present study, aqueous extracts of *P. venusta* leaves and flowers were analyzed, using both freeze-drying and hot-air oven-drying methods. When compared to the hydroalcoholic extracts reported by Silva et al. [33], the phenolic content of our aqueous extracts was second only to that of methanolic preparations. Consistent with findings by Shewale et al. [15], *P. venusta* flowers exhibited higher phenolic concentrations than leaves. Moreover, freeze-drying resulted in more efficient preservation of these compounds, demonstrating the influence of extraction and preservation methods on chemical stability and compound yield.

Phenolic compounds are widely recognized for their potent antioxidant properties, primarily through their capacity to scavenge reactive oxygen species (ROS) [34]. In line with this, the antioxidant potential of the *P. venusta* extracts was confirmed in our study, aligning with previous reports of antioxidant activity in various plant organs (leaves, roots, and flowers) using methanolic, ethanolic, and hexane-based extracts [14,15,18,29]. The discrepancy observed between the variation in total phenolic content and the relatively small differences in DPPH radical scavenging activity may be attributed to the fact that antioxidant activity is not determined solely by the total amount of phenolics. In particular, compounds such as caffeic and chlorogenic acids are potent radical scavengers, and their presence in both hot-air oven- and freeze-dried extracts may have contributed to similar DPPH responses, despite differences in overall phenolic concentration. Additionally, as we discuss below, possible synergistic or antagonistic interactions among the phytochemicals present can significantly influence antioxidant response, thereby reducing the variation observed in the DPPH assay compared to the total phenolic content. Notably, the freeze-dried extracts demonstrated a significantly higher antioxidant capacity, as determined by DPPH radical scavenging assays, highlighting the role of optimized processing methods in enhancing the bioactivity of plant-derived compounds. Among all samples tested, the freeze-dried flower extract exhibited the greatest antioxidant activity, corroborating the findings of Roy et al. [16]. These results are particularly important given the role of ROS in the pathogenesis of tissue injury, inflammation, and numerous chronic diseases [14].

Based on its superior phenolic content and antioxidant potential, the freeze-dried extract was selected for phytochemical characterization via ultra-high-performance liquid chromatography (UHPLC). This analysis identified several bioactive phenolics, including *trans*-cinnamic acid, gallic acid, *trans*-ferulic acid, chlorogenic acid, caffeic acid, p-coumaric acid, 3-O-methyl-quercetin, luteolin, quercetin, pelargonidin-3-O-glucoside, malvidin-3,5-diglycoside, peonidin-3-O-glucoside, and malvidin-3-O-glucoside. Among these, p-coumaric acid, more abundant in flowers, has been extensively reported for its antioxidant, antimicrobial, antiviral, antimutagenic, anticarcinogenic, and antidiabetic activities [35]. Chlorogenic acid, predominantly found in leaves, possesses hepatoprotective, antihypertensive, antibacterial, and anti-inflammatory properties [36]. Similarly, caffeic acid, also more concentrated in the leaves, shares many of the biological functions attributed to chlorogenic and p-coumaric acids, including antioxidant and anti-inflammatory effects [37]. *Trans*-ferulic acid, present in both organs, contributes additional pharmacological activities, including cardiovascular, neurological, and hematological protection, as well as anti-inflammatory and antidiabetic effects [38]. The coexistence of these compounds may promote synergistic interactions, amplifying the antioxidant effects observed in this study and reinforcing the therapeutic potential of *P. venusta* extracts. Although several distinct polyphenols were identified, comprehensive characterization through mass spectrometry (MS) or nuclear magnetic resonance (NMR) spectroscopy is expected to reveal additional constituents within the extracts, thereby strengthening the basis for future investigations.

The pharmacokinetic and toxicological predictions of the identified polyphenols reinforce their potential as bioactive agents, while simultaneously highlighting the necessity for further experimental validation. The Lipinski Rule of Five, proposed by Lipinski et al. in 1997 [39], is a widely used set of criteria that evaluates the drug-likeness of compounds based on molecular properties associated with favorable oral bioavailability. According to this rule, compounds that meet these criteria are more likely to exhibit good absorption and systemic distribution, making it a useful tool in early drug development. Most phenolic compounds identified in *P. venusta* complied with the rule, indicating favorable absorption and systemic distribution. However, malvidin-3-O-glucoside, mal-vidin-3,5-diglycoside, and peonidin-3-O-glucoside did not meet one or more criteria, suggesting limitations in gastrointestinal absorption or metabolic stability that warrant in vivo exploration.

To complement the Lipinski assessment, ADMET analyses were conducted. These predictions indicated that several compounds, such as 3-O-methyl-quercetin, luteolin, and various phenolic acids, exhibited favorable membrane permeability and transporter affinity. Notably, some compounds were predicted to cross the blood–brain barrier (BBB), highlighting their potential for neuroprotective or CNS-modulating applications. Nonetheless, rapid clearance and short plasma half-lives were also predicted, which could constrain therapeutic efficacy. These challenges may be addressed through pharmaceutical innovations such as pro-drugs, nanoformulations, and encapsulation technologies aimed at enhancing bioavailability and prolonging systemic retention. Safety assessments predicted a moderate hepatotoxic potential for *trans*-cinnamic acid, reinforcing the need for dose-dependent toxicity evaluations. Moreover, 3-O-methyl-quercetin and luteolin showed potential in silico carcinogenicity, immunogenicity, and mutagenicity, underscoring the importance of comprehensive toxicological assessments before clinical application. Despite these considerations, the lack of widespread predicted toxicity across most compounds supports their continued evaluation as therapeutic candidates. While previous in silico pharmacokinetic predictions provide valuable preliminary insights, particularly in early-stage screening, they do not replace empirical data. Future studies will be necessary to experimentally validate the predicted pharmacokinetic profiles and further confirm the biological relevance of the compounds identified.

Molecular docking has emerged as a key strategy in natural product drug discovery, offering insights into the interactions between bioactive molecules and specific molecular targets [40]. In this study, docking analyses were conducted to evaluate the binding of *P. venusta* polyphenols to Ki-67 and PCNA, proteins strongly associated with cell proliferation. This was motivated by our cytotoxicity results, which showed enhanced HaCaT cell proliferation upon treatment with *P. venusta* extract. While specific docking studies involving polyphenols and Ki-67 or PCNA remain scarce, recent work by Hassan et al. (2024) [41] demonstrated the ability of sesquiterpene lactones to bind these proteins and potentially exert antiproliferative effects. Other studies, such as those using *Moringa oleifera* extracts, support the utility of docking approaches for understanding phenolic–protein interactions [30]. Likewise, investigations of polyphenol binding to β-lactoglobulin have provided structural insights into the stability of such complexes [42]. Our results support the hypothesis that *P. venusta* polyphenols interact with proteins involved in DNA replication, repair, and mitosis, thereby potentially modulating cell cycle progression. Notably, our calculated binding energies and inhibition constants (Ki), ranging from nanomolar to low micromolar values, are consistent with values reported for polyphenol–CDK2 interactions, such as quercetin binding to CDK2 (−8.29 kcal/mol) described by Ullah et al. (2019) [43]. The strong binding affinity of *trans*-ferulic acid for conserved regions of PCNA, stabilized through hydrogen bonding and hydrophobic interactions, highlights its potential role in regulating proliferative responses. Despite its low lipophilicity, which limits oral bioavailability, functionalization strategies are under investigation to enhance the pharmacological utility of ferulic acid [44]. Similarly, the predicted strong interaction of caffeic acid with Ki-67 suggests a possible role in modulating physiological and pathological proliferation. Notably, while the phenethyl ester of caffeic acid has shown antiproliferative effects in various cancer models [45], treatment associating laccase and caffeic acid increased the proliferation of normal human epidermal melanocytes (NHEMs) and human embryonal carcinoma cells (NTERA-2) [46]. Although our docking results are preliminary and exploratory, they offer valuable insights that may guide future experimental validation and hypothesis-driven research.

Finally, we adopted an integrative approach to evaluate how the identified compounds might act collectively. While conventional pharmacological research often focuses on isolated phytochemicals, such reductionist strategies can overlook the complexity and therapeutic potential of whole-plant extracts. Notably, synergistic interactions among multiple constituents of medicinal plants have been well documented [47]. Our broader analysis of all identified polyphenols revealed that many of these compounds shared overlapping binding sites within PCNA and Ki-67, suggesting possible competitive interactions. However, specific polyphenols, including Malvidin-3,5-diglycoside, Peonidin-3-O-glucoside, Chlorogenic acid, and Gallic acid, were predicted to bind to distinct regions within PCNA. Similarly, Pelargonidin-3-O-glucoside and Pe-onidin-3-O-glucoside interacted with spatially separated pockets in Ki-67, indicating alternative and possibly complementary mechanisms of action. Phytochemicals can exert positive combinatory effects in various ways, such as enhancing each other’s bioavailability, stability, or cellular uptake. Synergistic and antagonistic relationships may occur both within extracts from a single plant and between components in polyherbal formulations. For instance, certain primary and secondary metabolites can improve the solubility of active compounds, inhibit their metabolic degradation, enhance membrane permeability, and modulate the delivery of bioactive agents [47,48,49,50].

The chemical complexity of crude plant extracts enables multiple bioactive molecules to act in concert, often resulting in synergistic effects at the molecular and cellular levels. Our in silico findings reinforce this view, demonstrating that polyphenols, if bioavailable, can simultaneously interact with multiple binding sites within a single protein. This suggests a multifaceted mode of action, far more intricate than what is typically considered when studying isolated compounds. Moreover, the ability of different polyphenols to compete for the same binding site adds a layer of dynamic molecular interplay that could influence biological outcomes. Consequently, focusing solely on isolated constituents may underestimate the full therapeutic potential of plant-derived treatments.

## 4. Materials and Methods

### 4.1. Material Collection and Production of Hot-Air Oven- and Freeze-Dried Extracts

Fresh flowers and leaves of *P. venusta* were collected from Lageado farm in Botucatu, São Paulo, Brazil (coordinates: 22°51′02.3″ S 48°26′09.5″ W). The plant material was sanitized using sodium hypochlorite solution and subsequently dried in a forced-air circulation oven (Marconi) at 45 °C for 12 h. After drying, the material was ground into a fine powder, which was referred to as the hot-air oven-dried extract.

To prepare the freeze-dried extract (also referred to as the lyophilized extract), 0.5 g of the powdered flower and leaf material was extracted with deionized water (100 °C) for 30 min in a water bath. The resulting mixture (0.05 g/mL) was allowed to cool to room temperature and centrifuged at 3500 rpm for 10 min [16]. The supernatant was then filtered through Whatman filter paper (Grade 2: 8 µm) to obtain the aqueous extract. This extract was distributed into beakers, placed on stainless steel trays, and frozen at −80 °C for 24 h. Subsequently, the samples were freeze-dried using a vacuum system operating at −30 °C and a flow rate of 10.2 m^3^/h for 48 h.

### 4.2. Preparation of Aqueous Extracts from Leaves and Flowers

To prepare the experimental solutions, 0.1 g of hot-air oven-dried or lyophilized leaf and flower samples was diluted in 100 mL of distilled water. The mixture was subjected to an ultrasonic bath for 10 min to facilitate the solubilization of bioactive compounds. After sonication, the samples were centrifuged at 3000 rpm for 10 min, and the supernatant was collected for subsequent analyses. The final concentration of the aqueous extracts was standardized at 1 mg/mL, which was used in all further assays.

### 4.3. Determination of Total Phenolic Compounds

The concentration of phenolic compounds in both the hot-air oven-dried and freeze-dried extracts was determined using the Folin–Ciocalteau method [51]. Aliquots of 0.5 mL of the extracts were added to 2.0 mL of diluted Folin–Ciocalteau (1:10; *v*/*v*). The solution was vortexed and allowed to stand in the dark for 5 min. Following this, 2.5 mL of a 7.5% sodium carbonate solution was added. The resulting solution was kept in the dark for 2 h. Absorbance was measured at 760 nm, and the results are expressed as mg of gallic acid equivalents per 100 g of sample.

### 4.4. Determination of Antioxidant Capacity by the DPPH Method

Antioxidant capacity was assessed using the DPPH (2,2-diphenyl-1picrylhydrazine) free radical scavenging method, according to the procedure described by Brand-Williams, Cuvelier, and Berset [52]. A DPPH solution (0.6 mM) was prepared and adjusted to an absorbance of 0.7 at 515 nm. Samples of 0.2 g of hot-air oven-dried or freeze-dried sample were dissolved in distilled water in a 25 mL volumetric flask and subjected to an ultrasonic bath for 10 min. After this, 2 mL of the resulting solution was mixed with 2 mL of the DPPH solution (labeled as a1). Simultaneously, the following two control mixtures were prepared: 2 mL of DPPH solution with 2 mL of ethanol (blank control, Ac) and 2 mL of the extract solution with 2 mL of distilled water (sample control, a2). All samples were incubated in the dark, and absorbance readings were taken after 3 h at 517 nm. The antioxidant capacity of the extracts from *P. venusta* flowers and leaves (both hot-air oven-dried and freeze-dried) was calculated according to the following formula, as proposed by Garcia et al. [53]:

$$Antioxidant\;capacity\;(\%)=\;\left[\left(1-\frac{a1-a2}{Ac}\right)\times 100\right]$$

a1 = absorbance of *P. venusta* extract + DPPH-solution; a2 = absorbance of *P. venusta* extract + water; Ac = absorbance of DPPH-solution + ethanol (control).

### 4.5. Total Antioxidant Activity by the Iron Reduction Method—FRAP

Antioxidant activity was assessed by the iron reduction method (FRAP), which was carried out according to the protocol described by Benzie et al. (1996) [54]. For each sample, 0.02 g of either hot-air oven-dried or freeze-dried extract was dissolved in water using a 10 mL volumetric flask and subjected to an ultrasonic bath for 10 min. Next, 150 µL of the resulting solution was mixed with 2850 µL of the FRAP reagent. The mixture was incubated in a water bath at 37 °C for 30 min, after which absorbance was measured at 593 nm using a spectrophotometer (Sinergy, Biotek Instruments, Winooski, VT, USA). A standard curve was generated using Trolox (99.9 to 549.4 µM), and the results are expressed as µM Trolox equivalents per gram of extract.

### 4.6. Cell Viability Assay

The cytotoxic effects of the extracts on cell viability were assessed using the human keratinocyte (HaCaT) cell line. Cells were cultured in flasks containing Dulbecco’s Modified Eagle Medium (DMEM) (Gibco, Paisley, UK), supplemented with 10% fetal bovine serum (FCS; Gibco), 100 IU/mL of penicillin, and 100 μg/mL of streptomycin (Gibco). Cultures were incubated at 37 °C in a humidified atmosphere containing 5% CO_2_ until they reached approximately 80% confluence. HaCaT cells were then seeded in 96-well plates at a density of 10,000 cells per well. After 24 h to allow for cell adhesion, the medium was replaced with fresh DMEM containing 100 µg/mL of hot-air oven-dried or freeze-dried *P. venusta* leaf or flower extracts, prepared in triplicate. The extract solutions were previously diluted in the culture medium, and 100 μL of the treatment solution was added to each well. Cells were exposed to the extracts for 24 and 48 h. Following incubation, the medium was removed, and cell viability was assessed using the Thiazolyl Blue Tetrazolium Bromide (MTT) assay. Cell viability is expressed as a percentage relative to untreated control cells (cells kept only in the culture medium).

### 4.7. Profile of Phenolic Compounds by Ultra-High-Performance Liquid Chromatography (UHPLC)

The identification and quantification of phenolic compounds present in the freeze-dried leaf and flower extracts of *P. venusta* were carried out using ultra-high-performance liquid chromatography (UHPLC-Ultimate 3000, Thermo Scientific, Waltham, MA, USA). Extract samples were filtered (PTFE, 0.45 m, Millipore, MA, USA) and injected (20 μL) into a UHPLC system (Ultimate 3000 BioRS, Dionex-Thermo Fisher Scientific Inc.) equipped with a cluster array detector diode (DAD), Luna^®^ 2.5 μm C18 column HST 2.0 × 50 mm (Phenomenex^®^, Torrance, CA, USA), and coupled with UV-Vis spectrophotometer. The run temperature was 39 °C and the flow rate was 0.6 mL/min. The mobile phase consisted of 0.85% phosphoric acid solution (solvent A) and 100% acetonitrile (solvent B). The gradient used was as follows: 0–2.5 min: 4% B; 2.5–7.5 min: 8% B; 7.5–15 min: 12% B; 15–18 min: 15% B; 18–20 min: 20% B; 20–21 min: 25% B; 21–22 min: 35% B; 22–24 min: 65% B; 24–25 min: 65% B; 25–25.5 min: 35% B; 25.5–26 min: 0% A; 26–27 min: 0% B. Calibration curves were prepared with commercial analytical standards (gallic acid, chlorogenic acid, pelargonidin-3-0-glycoside, caffeic acid, malvidin-3-5-diglycoside, peonidin-3-0-glycoside, malvidin-3-0-glycoside, ellagic acid, p-coumaric acid, *trans*-ferulic acid, 3-0-methyl quercetin, luteolin, resveratrol, quercetin, *trans*-cinnamic acid, and kaempferol; all from Sigma Aldrich), and based on their retention times, compound identification and quantification was performed at 280 nm, 320 nm, 360 nm, and 520 nm. Data are expressed as μg/mg. All analyses were performed in triplicate [55].

### 4.8. ADMET Predictions

The canonical SMILES structures of the identified flavonoids were used to predict their pharmacokinetic and toxicological properties—absorption, distribution, metabolism, excretion, and toxicity (ADMET)—using the Admetlab v2.0 platform (https://admetmesh.scbdd.com/; accessed on 5 February 2025). Additionally, the ProTox 3.0 tool (https://tox.charite.de/protox3/index.php?site=home; accessed on 5 February 2025) was employed to evaluate the potential hepatotoxicity, carcinogenicity, immunotoxicity, mutagenicity, and nutrition toxicity of the compounds.

### 4.9. In Silico Molecular Blind Docking

The target proteins selected for molecular docking simulations were human PCNA (Proliferating Cell Nuclear Antigen, PDB ID: 1VYM) and Ki-67 (PDB ID: 2AFF), which were downloaded from the Research Collaboratory for Structural Bioinformatics Protein Data Bank (RCSB PDB) database (https://www.rcsb.org/; accessed on 28 January 2025) [56]. Structurally, PCNA is a homotrimer comprising three identical subunits (A, B, and C), each with 261 amino acid residues; our docking analysis was performed using the isolated A subunit. Ki-67 comprises 3256 amino acid residues, and our analysis considered the structure with the lowest XPLOR target function value among the top 100 generated models [57]. The preparation of proteins for the molecular docking was conducted in the UCSF ChimeraX software (version 1.8) [58]. Water molecules and nonstandard residues were deleted.

The 3D structures of the polyphenols analyzed were downloaded from PubChem (https://pubchem.ncbi.nlm.nih.gov/, accessed on 29 January 2025) [28] and prepared in the Avogrado software (version 1.2.0) by adding hydrogens and optimizing the molecule geometry. The polyphenols were 3-O-Methylquercetin (CID:5280681), caffeic acid (CID:689043), chlorogenic acid (CID:1794427), gallic acid (CID: 370), luteolin (CID:5280445), Malvidin 3-O-glucoside (CID:443652), Malvidin-3,5-diglucoside (CID:441765), p-Coumaric acid (CID:637542), Pelargonidin-3-0-glucoside (CID:443648), Peonidin-3-0-glucoside (CID:443654), Quercetin (CID:5280343), *Trans*-cinnamic acid (CID:444539), and *Trans*-Ferulic Acid (CID:445858). The structures and isomeric simple molecular-input line-entry system (SMILE) are depicted in Table S1.

The next steps were performed in the Autodock Tools software (MGLTools-1.5.6). During this stage, any residual water molecules were deleted, and polar hydrogens and Kollman charges were attached to both protein and ligands. The polyphenol structures were treated to allow all rotatable bonds to remain flexible. For PCNA, the grid box was configured to encompass the entire A subunit, with dimensions of 126, 126, and 126 Å along the X-, Y-, and Z-axis. The grid center was set at the coordinates −41.633, −19.487, and 43.361, with a grid spacing of 0.464 Å. For Ki67, the grid box dimensions were 126 × 126 × 114 Å, centered at −1.224 (X), −0.513 (Y), and 1.492 (Z), with a grid spacing of 0.375 Å.

Docking parameters were defined as follows: genetic algorithm (GA) runs = 25; population size = 150; maximum number of energy evaluations = 25,000,000; and GA crossover mode = two-point. The Lamarckian Genetic Algorithm was employed to explore the conformational space, and the binding pose with the lowest binding energy was selected for further structural and interaction analysis. The resulting docking conformations were analyzed and visualized using UCSF ChimeraX and BIOVIA Discovery Studio Visualizer (v24.1.0).

### 4.10. Statistical Analysis

Data were initially assessed for normality using the Shapiro–Wilk test and for homogeneity of variances using Levene’s test. Statistical differences among groups were evaluated using one-way analysis of variance (ANOVA), followed by Tukey’s post hoc test for multiple comparisons. Differences in phenolic compound concentrations were analyzed using the Student’s *t*-test. Results were considered statistically significant at *p* < 0.05.

## 5. Conclusions

This study demonstrated that drying methods significantly affect the phenolic composition and bioactivity of *Pyrostegia venusta* extracts, with freeze-drying proving the most effective in preserving phenolics and antioxidant capacity. Caffeic and chlorogenic acids predominated in the leaves, while p-coumaric acid was more abundant in the flowers. Our approach aligns with green chemistry principles by employing eco-friendly extraction and sustainable drying, particularly freeze-drying, which maintains bioactive integrity without harmful byproducts. We further emphasize that the biological activity of plant extracts arises from synergistic interactions among constituents, not isolated compounds. This reinforces the need to evaluate plant-based therapies holistically and supports their development through sustainable and scientifically sound strategies. Future studies should explore pharmacokinetics and toxicity in vivo to validate their therapeutic potential.

## 6. Limitations

This study presents a preliminary yet relevant contribution to the understanding of the antioxidant and pro-proliferative potential of *Pyrostegia venusta* phenolic extracts. However, some limitations must be acknowledged. First, the identification and quantification of phenolic compounds were performed using UHPLC based on commercial standards, without the isolation or structural elucidation of new or unknown compounds. As such, the originality of the chemical findings is limited, and the study does not report novel molecules. Furthermore, although our UHPLC analysis was focused on freeze-dried extracts due to their higher stability and reliability, we recognize that including hot-air oven-dried extracts could have provided additional insights into the impact of drying on phenolic composition. Future studies should, therefore, address this point by directly comparing both drying methods at the chromatographic level. Additionally, although the molecular docking analyses with PCNA and Ki-67 provide valuable insights into the potential mechanisms of action of these compounds, these in silico predictions remain speculative in the absence of experimental validation, such as in vitro or in vivo assays specifically addressing these molecular targets. Similarly, the ADMET predictions offer only theoretical pharmacokinetic insights and should be interpreted with caution until corroborated by experimental pharmacological studies. Another limitation concerns the cytotoxicity assays, which were conducted without using a positive control. Lastly, this work did not include in vivo or clinical validations, thus warranting future studies on the therapeutic implications of our findings.

## Figures and Tables

**Table 1:** Phenolic compounds identified in the lyophilized extract of leaves and flowers of *P. venusta*.

			Quantification (µg/mg)		
R_t_	λ_max_	Tentative Identification	Leaves	Flowers	*p*-Value
11.33	360	3-O-methyl-quercetin	0.0292 ± 0.0004	0.5867 ± 0.0006	*p* < 0.0001
8.24	320	Caffeic acid	3.0728 ± 0.0105	0.1721 ± 0.0024	*p* < 0.0001
7.15	320	Chlorogenic acid	3.5582 ± 0.0213	2.1584 ± 0.0198	*p* < 0.0001
5.83	270	Gallic acid	0.0983 ± 0.0012	0.0215 ± 0.0015	*p* > 0.9999
11.47	360	Luteolin	0.2030 ± 0.0012	0.0404 ± 0.0014	*p* = 0.6951
8.71	520	Malvidin-3-O-glucoside	0.1752 ± 0.0538	0.5577 ± 0.2117	*p* = 0.0001
8.54	520	Malvidin-3-5-diglycoside	0.1016 ± 0.0329	0.3752 ± 0.1426	*p* = 0.0174
9.39	320	p-coumaric acid	1.3098 ± 0.0248	33.8937 ± 0.0466	*p* < 0.0001
8.14	520	Pelargonidin-3-O-glucoside	0.0952 ± 0.0215	0.0901 ± 0.0112	*p* > 0.9999
8.67	520	Peonidin-3-O-glucoside	0.1048 ± 0.0346	0.3194 ± 0.1215	*p* = 0.1460
11.57	360	Quercetin	0.5228 ± 0.0052	0.3078 ± 0.0008	*p* = 0.1441
12.65	270	*Trans*-cinnamic acid	0.004 ± 0.0001	0.2340 ± 1.1636	*p* = 0.0868
9.74	320	*Trans*-ferulic acid	1.7463 ± 0.0156	1.7756 ± 0.0279	*p* > 0.9999

Data were analyzed *t*-student test and expressed as mean ± SEM. R_t_—retention time (minutes); λ_max_—maximum absorbance (nm).

**Table 2:** Chemical properties of phenolic compounds identified in leaves and flowers of *P. venusta*.

Compound	M.W (g/mol)	H-Bond Acceptor	H-Bond Donor	logD	TPSA (Å)	Linpiski Rule
3-O-methyl-quercetin	316.26	7	4	1.75	120.36	Yes
Caffeic acid	180.16	4	3	0.93	77.76	Yes
Chlorogenic acid	354.31	9	6	−0.39	164.75	Yes
Gallic acid	170.12	5	4	0.21	97.99	Yes
Luteolin	286.24	6	4	1.73	111.13	Yes
Malvidin-3-O-glucoside	493.44	12	7	−0.90	191.67	No
Malvidin-3-5-diglycoside	655.58	17	10	−2.86	270.82	No
p-coumaric acid	164.16	3	2	1.26	57.53	Yes
Pelargonidin-3-O-glucoside	433.39	10	7	−0.84	173.21	Yes
Peonidin-3-O-glucoside	463.41	11	7	−0.69	182.44	No
Quercetin	302.24	7	5	1.23	131.36	Yes
*Trans*-cinnamic acid	148.16	2	1	1.79	37.30	Yes
*Trans*-ferulic acid	194.18	4	2	1.36	66.76	Yes

LogD: octanol/water partition coefficient at physiological pH. TPSA: Topological Polar Surface Area.

**Table 3:** Toxicity prediction parameters of phenolic compounds identified in leaves and flowers of *P. venusta*.

Compound	Parameters					
	LD50 (g/kg)	Hepato.	Carcino.	Immuno.	Muta.	Nutr.
3-0-methyl-quercetin	5.00	0.72-I	0.55-A	0.50-A	0.61-I	0.54-A
Caffeic acid	2.98	0.57-I	0.78-A	0.50-I	0.98-I	0.77-I
Chlorogenic acid	5.00	0.72-I	0.68-I	0.99-A	0.93-I	0.64-I
Gallic acid	2.00	0.61-I	0.56-A	0.99-I	0.94-I	0.83-I
Luteolin	3.91	0.69-I	0.68-A	0.97-I	0.51-A	0.63-A
Malvidin-3-0-glucoside	5.00	0.81- I	0.89-I	0.95-A	0.74-I	0.51-I
Malvidin-3-5-diglycoside	5.00	0.78-I	0.87-I	0.94-A	0.73-I	0.52-I
p-coumaric acid	2.85	0.51-I	0.5-A	0.91-I	0.93-I	0.89-I
Pelargonidin-3-0-glucoside	5.00	0.76-I	0.86-I	0.56-I	0.72-I	0.53-A
Peonidin-3-0-glucoside	5.00	0.82-I	0.87-I	0.89-A	0.65-I	0.51-I
Quercetin	0.16	0.69-I	0.68-A	0.87-I	0.51-A	0.63-A
*Trans*-cinnamic acid	2.5	0.54-A	0.82-I	0.95-I	0.96-I	0.92-I
*Trans*-ferulic acid	1.77	0.51-I	0.61-I	0.91-A	0.96-I	0.82-I

The values refer to the probability of the compound being Active (A) or Inactive (I). Hepato.: hepatotoxicity; Carcino.: carcinogenicity, Immuno.: immunotoxicity; Muta.: mutagenicity; Nutr.: Nutritional toxicity.

**Table 4:** Molecular docking results of phenolic compounds identified in leaves and flowers of *P. venusta*.

Polyphenol (Ligand)	PCNA		Ki-67	
	Binding Affinity	Inhibition Constant	Binding Affinity	Inhibition Constant
3-0-methyl-quercetin	−10.48 Kcal/mol	20.94 nM	−10.23 Kcal/mol	31.72 nM
Caffeic acid	−7.64 Kcal/mol	2.50 uM	−8.15 Kcal/mol	1.06 uM
Chlorogenic acid	−11.25 Kcal/mol	5.67 nM	−6.50 Kcal/mol	11.11 uM
Gallic acid	−7.70 Kcal/mol	2.25 uM	−7.52 Kcal/mol	3.07 uM
Luteolin	−5.66 Kcal/mol	70.82 uM	−6.06 Kcal/mol	35.92 uM
Malvidin-3-0-glucoside	−12.09 Kcal/mol	1.37 nM	−10.39 Kcal/mol	24.10 nM
Malvidin-3-5-diglycoside	−9.21 Kcal/mol	177.22 nM	−11.20 Kcal/mol	6.16 nM
p-coumaric acid	−7.54 Kcal/mol	3.00 uM	−7.43 Kcal/mol	3.59 uM
Pelargonidin-3-0-glucoside	−11.55 Kcal/mol	3.41 nM	−10.20 Kcal/mol	33.57 nM
Peonidin-3-0-glucoside	−11.25 Kcal/mol	5.64 nM	−10.47 Kcal/mol	21.23 nM
Quercetin	−10.17 Kcal/mol	35.3 nM	−9.66 Kcal/mol	82.32 nM
*Trans*-cinnamic acid	−5.57 Kcal/mol	83.04 uM	−5.08 Kcal/mol	189.35 uM
*Trans*-ferulic acid	−8.16 Kcal/mol	1.05 uM	−7.84 Kcal/mol	1.79 uM

## Data Availability

Data are contained within the article and Supplementary Materials.

## Supplementary Materials

The following supporting information can be downloaded at: https://www.mdpi.com/article/10.3390/ph18091315/s1, Table S1: Phenolic compounds and their structural characteristics.
