# Supplementary material for: Effect of Drying Methods on Bioactivity of Pyrostegia venusta Extracts: Antioxidant Assays, Cytotoxicity, and Computational Approaches
Source: Pharmaceuticals (Basel). 2025 Sep 2;18(9):1315. doi: 10.3390/ph18091315 (PMC12472987; doi:10.3390/ph18091315)
Supplement: Supplementary file 1 [file pharmaceuticals-18-01315-s001.zip › pharmaceuticals-3825486-supplementary.pdf]

Supplementary Table

Effect of Drying Methods on the Bioactivity of *Pyrostegia venusta* Extracts: Antioxidant assays, Cytotoxicity and Computational Approaches

Table S1. Phenolic compounds and their structural characteristics.

Compound	2D Structure	SMILES
3-O-Methylquercetin (PubChem CID:5280681)	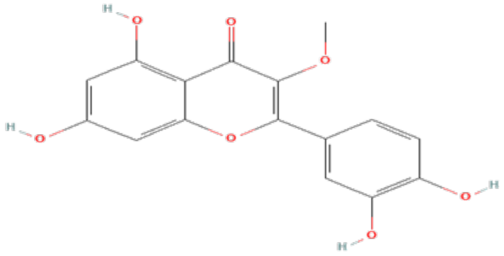	COC1=C(OC2=CC(=CC(=C2C1=O)O)O)C3=CC(=C(C=C3)O)O
Caffeic Acid (PubChem CID:689043)	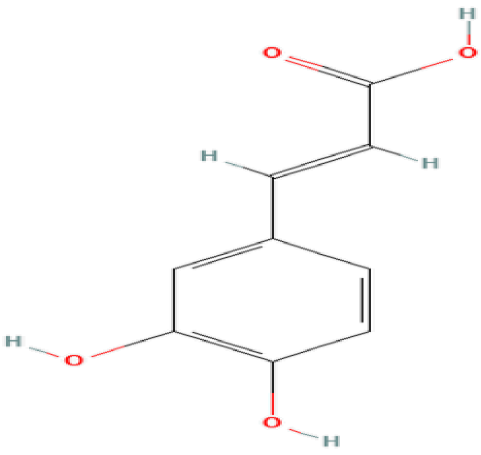	C1=CC(=C(C=C1/C=C/C(=O)O)O)O
Chlorogenic Acid (PubChem CID:1794427)	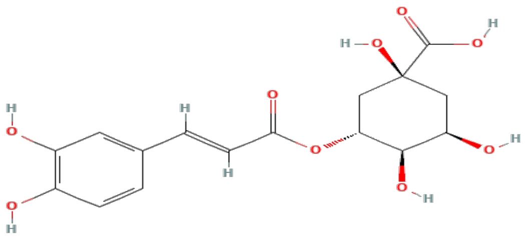	C1[C@H]([C@H]([C@@H](C[C@@]1(C(=O)O)O)O)OC(=O)/C=C/C2=CC(=C(C=C2)O)O)O)O

Gallic Acid (PubChem CID:370)	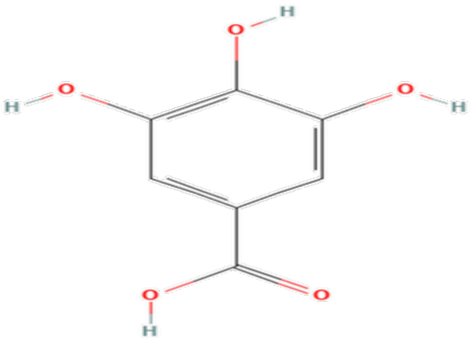	C1=C(C=C(C(=C1O)O)O)C(=O)O
Luteolin (PubChem CID:5280445)	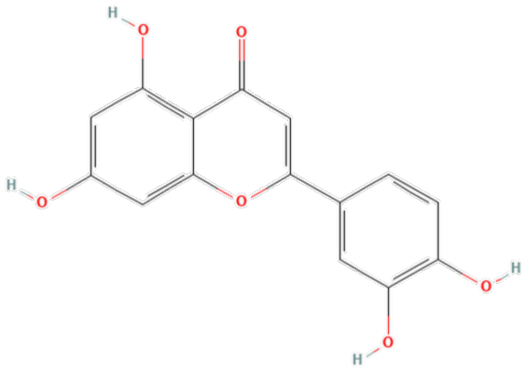	C1=CC(=C(C=C1C2=CC(=O)C3=C(C=C(C=C3O2)O)O)O)O
Malvidin 3-O-glucoside (PubChem CID:443652)	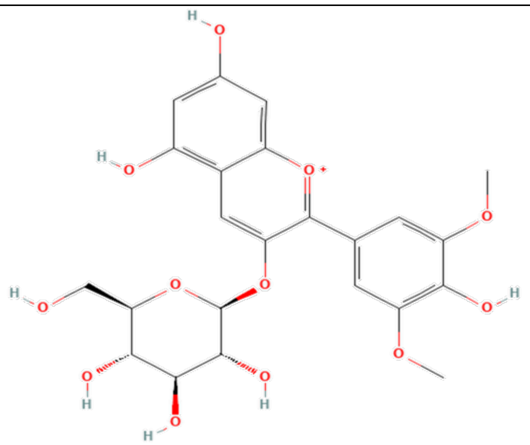	COC1=CC(=CC(=C1O)OC)C2=[O+]C3=CC(=CC(=C3C=C2O[C@H]4[C@@H]([C@H]([C@@H]([C@H](O4)CO)O)O)O)O)O
Malvidin-3-5-diglycoside (PubChem CID:441765)	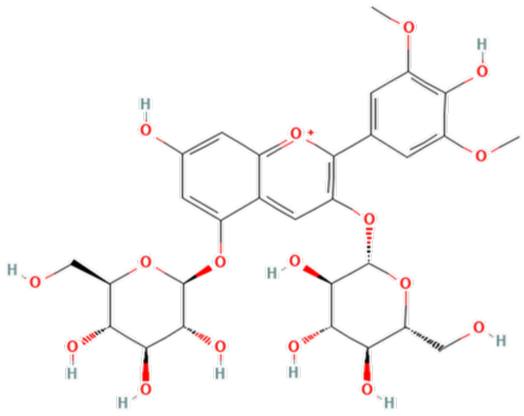	COC1=CC(=CC(=C1O)OC)C2=C(C=C3C(=CC(=CC3=[O+]2)O)O[C@H]4[C@@H]([C@H]([C@@H]([C@H](O4)CO)O)O)O)O[C@H]5[C@@H]([C@H]([C@@H]([C@H](O5)CO)O)O)O

p-Coumaric acid (PubChem CID:637542)	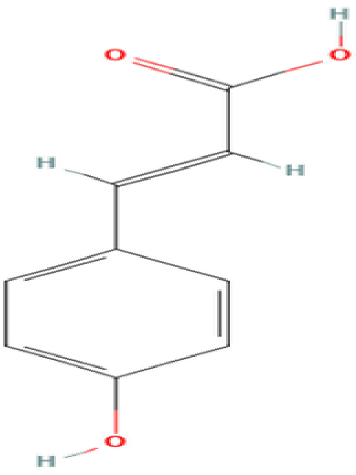	C1=CC(=CC=C1/C=C/C(=O)O)O
Pelargonidin-3-0-glucoside (PubChem CID:443648)	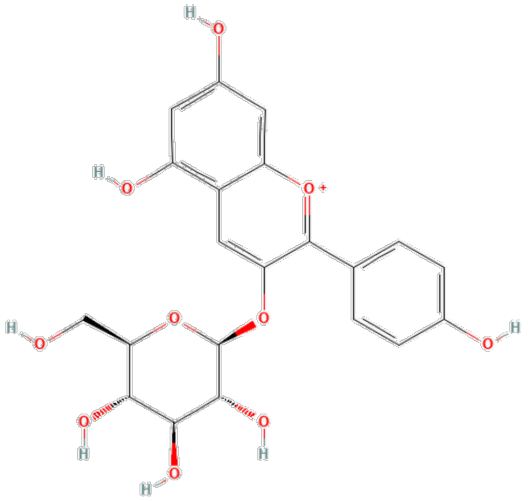	C1=CC(=CC=C1C2=[O+]C3=CC(=CC(=C3C=C2O[C@H]4[C@@H]([C@H]([C@@H]([C@H](O4)CO)O)O)O)O)O)O
Peonidin-3-0-glucoside (PubChem CID:443654)	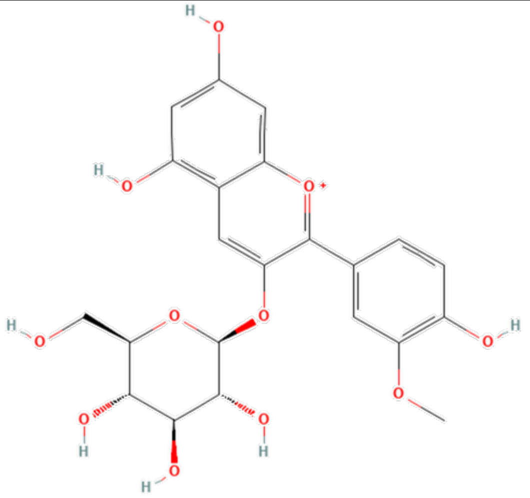	COC1=C(C=CC(=C1)C2=[O+]C3=CC(=CC(=C3C=C2O[C@H]4[C@@H]([C@H]([C@@H]([C@H](O4)CO)O)O)O)O)O)O

Quercetin<br>(PubChem CID:5280343)	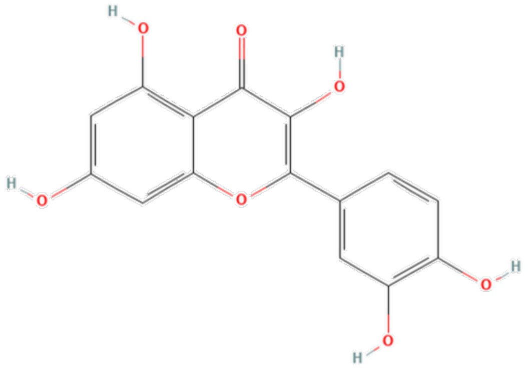	C1=CC(=C(C=C<br>1C2=C(C(=O)C<br>3=C(C=C(C=C3<br>O2)O)O)O)O)O
Trans-cinnamic acid<br>(PubChem CID:444539)	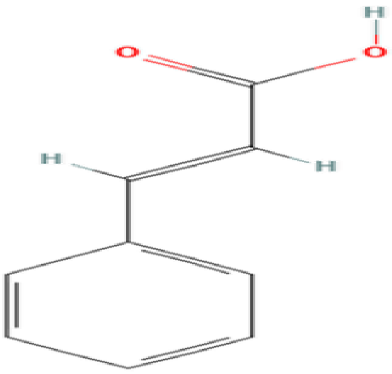	C1=CC=C(C=C1<br>)/C=C/C(=O)O
Trans-Ferulic Acid<br>(PubChem CID:445858)	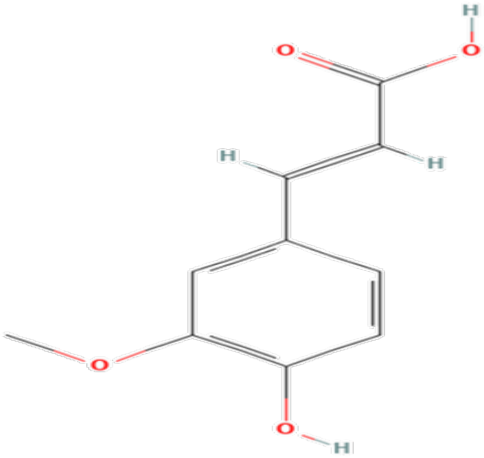	COC1=C(C=CC(<br>=C1)/C=C/C(=<br>O)O)O
